# Supplementary material for: Is the Validity of Logistic Regression Models Developed with a National Hospital Database Inferior to Models Developed from Clinical Databases to Analyze Surgical Lung Cancers?
Source: Cancers (Basel). 2024 Feb 9;16(4):734. doi: 10.3390/cancers16040734 (PMC10886576; doi:10.3390/cancers16040734)
Supplement: Supplementary file 1 [file cancers-16-00734-s001.zip › cancers-2665510-supplementary.pdf]

cancers MDPI

Supplementary Materials

Table S1. Comparison of patient and hospital characteristics according to 30-day mortality in training clinical database Epithor (n=10 516).

	No n=10,289(97.84%)	Yes n=227(2.16%)	p-value*
Age (year)	64.7 (9.5)	67.0 (9.6)	<0.001
Gender			
Male	6,467 (97.2%)	185 (2.8%)	<0.001
Female	3,822 (98.9%)	42 (1.1%)	
Body Mass Index (kg/m2)	25.571 (4.7)	25.157 (4.0)	0.422
Performance status			
0	5,541 (98.6%)	79 (1.4%)	<0.001
1	4,005 (97.4%)	106 (2.6%)	
2	743 (94.6%)	42 (5.4%)	
Dyspnea score			
0	6,467 (98.4%)	108 (1.6%)	<0.001
1	2,799 (97.5%)	73 (2.5%)	
2	875 (97.1%)	26 (2.9%)	
3	148 (88.1%)	20 (11.9%)	
FEV1	87.9 (18.8)	82.1 (17.7)	<0.001
Gold score			
0	9,002 (98%)	180 (2%)	<0.001
1	501 (97.5%)	13 (2.5%)	
2	725 (96.3%)	28 (3.7%)	
3	59 (92.2%)	5 (7.8%)	
4	2 (66.7%)	1 (33.3%)	
ASA score			
1	1,807 (99.1%)	16 (0.9%)	<0.001
2	4,988 (98.2%)	91 (1.8%)	
3	3,494 (96.7%)	120 (3.3%)	
Pulmonary resection			
Wedge	967 (96.9%)	31 (3.1%)	<0.001
Segmentectomy	1,015 (98.7%)	13 (1.3%)	
Lobectomy	7,380 (98.1%)	141 (1.9%)	
Bilobectomy	348 (96.1%)	14 (3.9%)	
Pneumonectomy	579 (95.4%)	28 (4.6%)	
Extended resection			
No	10,046 (97.9%)	218 (2.1%)	0.118
Yes	243 (96.4%)	9 (3.6%)	
Sleeve			
No	10,075 (97.9%)	214 (2.1%)	<0.001
Yes	214 (94.3%)	13 (5.7%)	
Approach			
Thoracotomy	5,374 (97.4%)	143 (2.6%)	0.001
VATS/robot	4,915 (98.3%)	84 (1.7%)	
T			
T1a	2,210 (98.4%)	36 (1.6%)	<0.001
T1b	1,637 (98.6%)	23 (1.4%)	
T1c	282 (99.3%)	2 (0.7%)	

T2a	2,131 (98.1%)	42 (1.9%)	
T2b	592 (97%)	18 (3%)	
T3	1,267 (95.8%)	56 (4.2%)	
T4	458 (93.9%)	30 (6.1%)	
Missing	1,712 (98.8%)	20 (1.2%)	
N			
N0	6,492 (98.2%)	120 (1.8%)	<0.001
N1	972 (96.3%)	37 (3.7%)	
N2	1,098 (95.6%)	50 (4.4%)	
Missing	1,727 (98.9%)	20 (1.1%)	
M			
M0	8,191 (97.8%)	187 (2.2%)	<0.001
M1	287 (92.9%)	22 (7.1%)	
Missing	1,811 (9.0%)	18 (1.0%)	
Preoperative treatment			
No	10,184 (97.9%)	222 (2.1%)	0.083
Yes	105 (95.5%)	5 (4.5%)	
Logarithm (hospital volume)	5.5 (0.8)	5.5 (0.8)	0.586
Type of hospitals			
Non academic	1,078 (98.1%)	21 (1.9%)	0.149
Academic	5,837 (97.7%)	137 (2.3%)	
Private non-profit	628 (99.1%)	6 (0.9%)	
Private for profit	2,746 (97.8%)	63 (2.2%)	
Alcohol use disorder			
No	9,727 (97.9%)	208 (2.1%)	0.058
Yes	562 (96.7%)	19 (3.3%)	
Smoker			
No	7,803 (97.9%)	170 (2.1%)	0.741
Yes	2,486 (97.8%)	57 (2.2%)	
Toxicology			
No	10,065 (97.9%)	220 (2.1%)	0.357
Yes	224 (97%)	7 (3.0%)	
Number of comorbidities			
0	1,655 (98.8%)	20 (1.2%)	0.001
1	2,867 (98.3%)	51 (1.7%)	
2	2,706 (97.4%)	73 (2.6%)	
≥3	3,061 (97.4%)	83 (2.6%)	

* Categorical variables were compared with a Chi-square test and quantitative variables were compared using a Student's t-test.

Table S2. Comparison of patient and hospital characteristics according to 30-day mortality in validation data clinical database Epithor.

	No<br>n=4,414 (98%)	Yes<br>n=93 (2%)	p-value*
Age (year)	64.6 (9.7)	68.6 (9.0)	<0.001
Gender			
Male	2,841 (97.3%)	80 (2.7%)	<0.001
Female	1,573 (99.2%)	13 (0.8%)	
Body Mass Index (kg/m2)	25.6 (4.7)	24.7 (4.1)	0.082

Performance Status			
0	2,343 (98.8%)	29 (1.2%)	<0.001
1	1,746 (97.2%)	51 (2.8%)	
2	325 (96.2%)	13 (3.8%)	
Dyspnea score			
0	2,761 (98.2%)	50 (1.8%)	<0.001
1	1,204 (98.4%)	19 (1.6%)	
2	388 (95.6%)	18 (4.4%)	
3	61 (91%)	6 (9.0%)	
FEV (l)	87.6 (19.1)	80.3 (20.6)	<0.001
Gold score			
0	3,885 (98.1%)	76 (1.9%)	0.470
1	200 (97.1%)	6 (2.9%)	
2	293 (96.7%)	10 (3.3%)	
3	35 (97.2%)	1 (2.8%)	
4	1 (100%)	0 (0%)	
ASA score			
1	751 (99.2%)	6 (0.8%)	0.009
2	2,162 (98.0%)	45 (2.0%)	
3	1,501 (97.3%)	42 (2.7%)	
Pulmonary resection			
Wedge	416 (98.1%)	8 (1.9%)	<0.001
Segmentectomy	417 (98.1%)	8 (1.9%)	
Lobectomy	3,153 (98.3%)	53 (1.7%)	
Bilobectomy	160 (96.4%)	6 (3.6%)	
Pneumonectomy	268 (93.7%)	18 (6.3%)	
Extended resection			
No	4,312 (98.0%)	88 (2.0%)	0.055
Yes	102 (95.3%)	5 (4.7%)	
Sleeve			
No	4,307 (97.9%)	91 (2.1%)	0.865
Yes	107 (98.2%)	2 (1.8%)	
Approach			
Thoracotomy	2,294 (97.3%)	63 (2.7%)	0.003
VATS	2,120 (98.6%)	30 (1.4%)	
T			
T1a	934 (98.3%)	16 (1.7%)	0.008
T1b	665 (98.1%)	13 (1.9%)	
T1c	138 (98.6%)	2 (1.4%)	
T2a	922 (98.5%)	14 (1.5%)	
T2b	243 (98.4%)	4 (1.6%)	
T3	558 (96.0%)	23 (4.0%)	

T4	203 (95.8%)	9 (4.2%)	
Missing	751 (98.4%)	12 (1.6%)	
N			
N0	2,733 (98.3%)	47 (1.7%)	0.004
N1	444 (96.5%)	16 (3.5%)	
N2	483 (96.4%)	18 (3.6%)	
Missing	754 (98.4%)	12 (1.6%)	
M			
M0	3,499 (98.0%)	73 (2%)	0.129
M1	130 (95.6%)	6 (4.4%)	
Missing	785 (98.2%)	14 (1.8%)	
Preoperative treatment			
No	4,379 (97.9%)	92 (2.1%)	0.762
Yes	35 (97.2%)	1 (2.8%)	
Logarithm (hospital volume)	5.5 (0.8)	5.4 (0.9)	0.745
Type of hospitals			
Non academic	463 (97.5%)	12 (2.5%)	0.250
Academic	2,457 (97.9%)	53 (2.1%)	
Private non-profit	255 (99.6%)	1 (0.4%)	
Private for profit	1,239 (97.9%)	27 (2.1%)	
Alcohol use disorder			
No	4,192 (98.1%)	82 (1.9%)	0.003
Yes	222 (95.3%)	11 (4.7%)	
Smoker			
No	3300 (98.0%)	66 (2.0%)	0.405
Yes	1,114 (97.6%)	27 (2.4%)	
Toxicology			
No	4,321 (97.9%)	91 (2.1%)	0.977
Yes	93 (97.9%)	2 (2.1%)	
Number of comorbidities			
0	735 (99.5%)	4 (0.5%)	<0.001
1	1,230 (98.4%)	20 (1.6%)	
2	1,135 (97.8%)	25 (2.2%)	
≥3	1,314 (96.8%)	44 (3.2%)	

* Categorical variables were compared with a Chi-square test and quantitative variables were compared using a Student's t-test.

Table S3. Comparison of patient and hospital characteristics according to 30-day mortality in the training data hospital database (n=10 516).

	No<br>10,233 (97.3%)	Yes<br>283 (2.7%)	p-value*
Age (years)	65.5 (9.6)	69.6 (9.2)	<0.001
Logarithm (hospital volume)	4.9 (0.9)	4.8 (0.9)	0.091
Pulmonary disease			

No	7,118 (98.9%)	79 (1.1%)	<0.001
Yes	3,115 (93.9%)	204 (6.1%)	
Heart disease			
No	8,672 (97.9%)	183 (2.1%)	<0.001
Yes	1,561 (94.0%)	100 (6.0%)	
Peripheral vascular disease			
No	9,332 (97.6%)	225 (2.4%)	<0.001
Yes	901 (94.0%)	58 (6.0%)	
Neurological disease			
No	9,832 (97.5%)	254 (2.5%)	<0.001
Yes	401 (93.3%)	29 (6.7%)	
Liver disease			
No	10,152 (97.5%)	265 (2.5%)	<0.001
Yes	81 (81.8%)	18 (18.2%)	
Renal disease			
No	9,962 (97.5%)	258 (2.5%)	<0.001
Yes	271 (91.6%)	25 (8.4%)	
Metabolic disease			
No	8,924 (97.3%)	243 (2.7%)	0.505
Yes	1,309 (97.0%)	40 (3%)	
Anemia			
No	8,852 (97.7%)	207 (2.3%)	<0.001
Yes	1,381 (94.8%)	76 (5.2%)	
Infectious disease			
No	10,173 (97.4%)	267 (2.6%)	<0.001
Yes	60 (78.9%)	16 (21.1%)	
Other disease			
No	5,973 (98.0%)	120 (2.0%)	<0.001
Yes	4,260 (96.3%)	163 (3.7%)	
Other treatment			
No	9,181 (97.5%)	235 (2.5%)	<0.001
Yes	1,052 (95.6%)	48 (4.4%)	
Extended resection			
No	9,179 (97.6%)	221 (2.4%)	<0.001
Yes	1,054 (94.4%)	62 (5.6%)	
Sleeve			
No	10,001 (97.4%)	268 (2.6%)	<0.001
Yes	232 (93.9%)	15 (6.1%)	
Gender			
Male	6,376 (96.4%)	236 (3.6%)	<0.001
Female	3,857 (98.8%)	47 (1.2%)	
Approach			
Thoracotomy	5,855 (96.3%)	222 (3.7%)	<0.001
VATS/robot	4,378 (98.6%)	61 (1.4%)	
Pulmonary resection			
Limited resection	1,651 (97.5%)	42 (2.5%)	<0.001
Lobectomy	7,700 (97.9%)	166 (2.1%)	
Bilobectomy	308 (93.3%)	22 (6.7%)	
Pneumonectomy	574 (91.5%)	53 (8.5%)	
Charlson modified score			
0	4,701 (98.1%)	93 (1.9%)	<0.001

1	992 (98.5%)	15 (1.5%)	
2	1,062 (96.7%)	36 (3.3%)	
>=3	3,478 (96.2%)	139 (3.8%)	
Type of hospitals			
Non academic	1,256 (96.9%)	40 (3.1%)	0.046
Academic	4,491 (97.4%)	121 (2.6%)	
Private non-profit	1,310 (98.3%)	22 (1.7%)	
Private for profit	3,176 (96.9%)	100 (3.1%)	

* Categorical variables were compared with a Chi-square test and quantitative variables were compared using a Student's t-test.

Table S4. Comparison of patient and hospital characteristics according to 30-day mortality in the validation data hospital database (n=4 507).

	No<br>4,388 (97.36%)	Yes<br>119 (2.64%)	p-value*
Age (years)	65.3 (9.7)	68.6 (8.8)	<0.001
Logarithm (Hospital volume)	5.0 (1.0)	4.7 (1.0)	0.011
Pulmonary disease			
No	2,968 (98.7%)	40 (1.3%)	<0.001
Yes	1,420 (94.7%)	79 (5.3%)	
Heart disease			
No	3,740 (97.8%)	85 (2.2%)	<0.001
Yes	648 (95.0%)	34 (5.0%)	
Peripheral vascular disease			
No	3,987 (97.6%)	96 (2.4%)	<0.001
Yes	401 (94.6%)	23 (5.4%)	
Neurological disease			
No	4,207 (97.6%)	105 (2.4%)	<0.001
Yes	181 (92.8%)	14 (7.2%)	
Liver disease			
No	4,357 (97.6%)	105 (2.4%)	<0.001
Yes	31 (68.9%)	14 (31.1%)	
Renal disease			
No	4,272 (97.6%)	107 (2.4%)	<0.001
Yes	116 (90.6%)	12 (9.4%)	
Metabolic disease			
No	3,869 (97.7%)	91 (2.3%)	<0.001
Yes	519 (94.9%)	28 (5.1%)	
Anemia			
No	3,804 (97.9%)	82 (2.1%)	<0.001
Yes	584 (94.0%)	37 (6.0%)	
Infectious disease			
No	4,366 (97.4%)	115 (2.6%)	<0.001
Yes	22 (84.6%)	4 (15.4%)	
Other disease			
No	2,543 (98.0%)	51 (2.0%)	0.001
Yes	1,845 (96.4%)	68 (3.6%)	
Other treatment			
No	3,953 (97.7%)	94 (2.3%)	<0.001
Yes	435 (94.6%)	25 (5.4%)	
Extended resection			

No	3,938 (97.5%)	100 (2.5%)	0.044
Yes	450 (95.9%)	19 (4.1%)	
Sleeve			
No	4,293 (97.4%)	115 (2.6%)	0.380
Yes	95 (96.0%)	4 (4.0%)	
Gender			
Male	2,665 (96.3%)	101 (3.7%)	<0.001
Female	1,723 (99.0%)	18 (1.0%)	
Approach			
Thoracotomy	2,458 (96.5%)	88 (3.5%)	<0.001
VATS/robot	1,930 (98.4%)	31 (1.6%)	
Pulmonary resection			
Limited resection	689 (97.6%)	17 (2.4%)	<0.001
Lobectomy	3,345 (98.1%)	66 (1.9%)	
Bilobectomy	112 (90.3%)	12 (9.7%)	
Pneumonectomy	242 (91.0%)	24 (9.0%)	
Charlson modified score			
0	1,971 (98.2%)	36 (1.8%)	0.005
1	395 (97.8%)	9 (2.2%)	
2	501 (96.9%)	16 (3.1%)	
>=3	1,521 (96.3%)	58 (3.7%)	
Type of hospitals			
Non academic	552 (96.3%)	21 (3.7%)	0.255
Academic	1,993 (97.6%)	49 (2.4%)	
Private non-profit	551 (98.0%)	11 (2.0%)	
Private for profit	1,292 (97.1%)	38 (2.9%)	

* Categorical variables were compared with a Chi-square test and quantitative variables were compared using a Student's t-test.

Table S5. Evaluation of performance of logistic regression model of clinical database without missing TNM categories.

	Epithor Clinical database	
	Training data (n=10516)	Validation data (n=4507)
Performance measures		
Brier score	0.02	0.02
Brier max	0.021	0.02
Brier scaled	0.027	0.08
Discriminative ability		
AUC ROC	0.77 [0.74- 0.8]	0.74 [0.69 - 0.78]
Concordance statistic		0.74
Discrimination slope		0.026
Calibration		
Hosmer-Lemeshow test (X^2) (P-value)	13.87 (0.08)	9.36 (0.4)
ICI		0.003
E50		0.002

E90	0.005
Emax	0.58
Abs Calibration Error*	0.005
Unreliability p value	0.2

ICI: Integrated Calibration Index, *Mean Absolute calibration error.

Table S6. Logistic model regression developed with training data from the Epithor clinical database without missing TNM categories.

	Coef	S.E.	Wald test	P value
Intercept	-8.0382	1.6889	-4.76	<0.0001
FEV 1*	0.0192	0.0133	1.44	0.1495
FEV 2	-0.0819	0.0414	-1.98	0.0481
FEV 3	0.2986	0.1606	1.86	0.0630
Age	0.0224	0.0079	2.82	0.0049
Body Mass Index**				
BMI: X/10	1.3302	0.7964	1.67	0.0949
BMI : X^3	-0.0790	0.0371	-2.13	0.0331
Performance status (ref=0)				
1	0.3402	0.1558	2.18	0.0290
⩾ 2	0.7261	0.2247	3.23	0.0012
Dyspnea score ⩾2	1.3253	0.2851	4.65	<0.0001
Gold score ⩾3	0.3136	0.2118	1.48	0.1387
Pneumonectomy	0.4427	0.2342	1.89	0.0587
Sleeve	0.9454	0.3104	3.05	0.0023
VATS	-0.0754	0.1526	-0.49	0.6209
Extended resection	0.2564	0.3658	0.70	0.4834
TNM stage				
T (ref=T1)				
T2	0.3348	0.1767	1.89	0.0581
T3	0.8123	0.1920	4.23	<0.0001
T4	1.0314	0.2419	4.26	<0.0001
N (ref=N0 N1)				
N2	0.1962	0.2001	0.98	0.3268
M (ref=M0)				
M1	0.8859	0.2572	3.44	0.0006
Female	-0.7076	0.1802	-3.93	<0.0001
ASA score (ref=0 – 1)				
ASA score 2	0.3974	0.2776	1.43	0.1522
ASA score 3	0.6743	0.2818	2.39	0.0167
Comorbidity score ⩾3	0.2718	0.1475	1.84	0.0653

* Restricted cubic splines,** fractional polynomial.

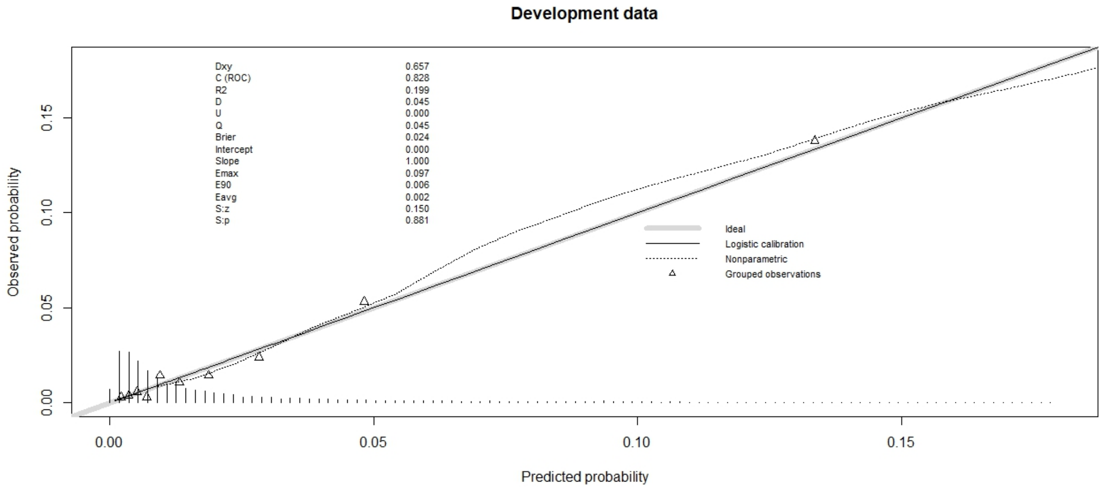

Figure S1. Calibration plot of observed mortality vs predicted mortality. Calibration for training data of hospital database (n=10 516).

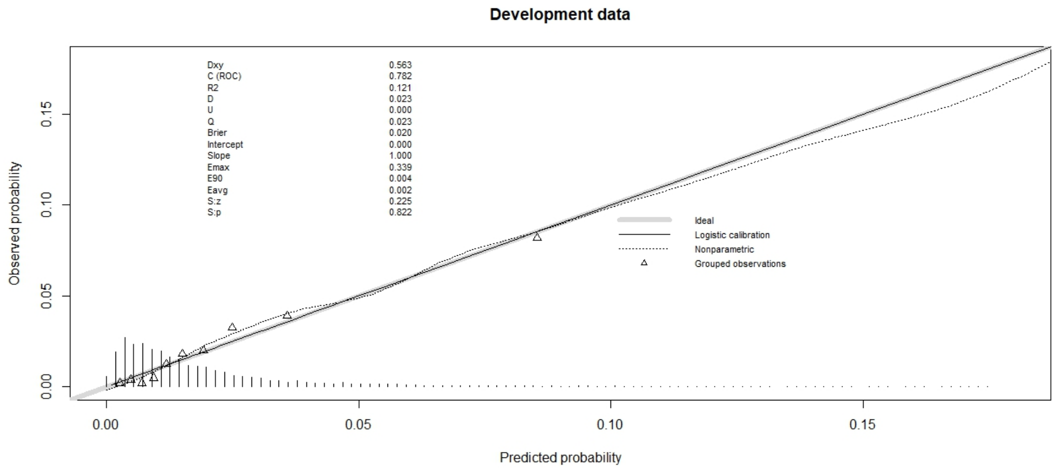

Figure S2. Calibration plot of observed mortality vs predicted mortality. Calibration for training data of Epithor clinical database (n=10 516).
